# Synaptic Proteostasis in Health and Disease

**DOI:** 10.1111/jnc.70565

**Published:** 2026-10-08

**Authors:** Paul G. Donlin‐Asp, Emma L. Clayton

**Affiliations:** ^1^ Simons Initiative for the Developing Brain, Institute for Neuroscience and Cardiovascular Research University of Edinburgh Edinburgh UK; ^2^ UK Dementia Research Institute at King's College London UK; ^3^ Department of Basic and Clinical Neuroscience, Institute of Psychiatry Psychology & Neuroscience King's College London UK

**Keywords:** autophagy, local translation, neurodegenerative disease, neurodevelopmental disease, proteostasis, synapse

## Abstract

Neurons rely on tightly coordinated mechanisms of protein synthesis and degradation to maintain cellular homeostasis, a process known as proteostasis. Given their highly polarised and compartmentalised nature, regulation of proteostasis is especially important at neuronal synapses, which are spatially distant from the soma yet require rapid on‐demand adaptation of the synaptic proteome. Neurons meet their demand for synaptic proteostasis through localised mRNA transport and translation, alongside specialised pathways for protein degradation. Recent advances reveal that thousands of transcripts localise to distal neuronal compartments and that their distribution may be shaped by RNA stability, RNA dynamics, and organelle‐hitchhiking transport mechanisms. In parallel, processes such as synaptic autophagy and endolysosomal trafficking are crucial for maintaining synaptic structure and neurotransmission. Disruptions in these finely balanced mechanisms are a common underlying feature of various neurological disorders, including fragile X syndrome, amyotrophic lateral sclerosis, Parkinson's disease, and Alzheimer's disease. This overview highlights key milestones and remaining questions in synaptic proteostasis, focusing on how local protein synthesis and degradation work together to preserve synaptic integrity and how their dysregulation can lead to disease.

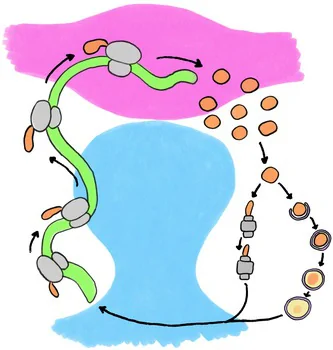

## Graphical abstract

The localised creation and degradation of proteins at the pre and postsynaptic terminal are processes that are crucial for neuronal homeostasis. Synaptic proteostasis pathways are frequently reported as dysregulated in neurodevelopmental and neurodegenerative disorders.

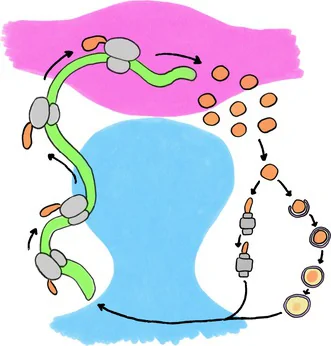

**Abbreviations**
AD: Alzheimer's disease
ALS: amyotrophic lateral sclerosis
APP: amyloid precursor protein
ASD: autism spectrum disorder
ATG: autophagy‐related genes
CNV: copy number variation
FAD: familial Alzheimer's disease
FMRP: fragile X messenger ribonucleoprotein
FTD: frontotemporal dementia
FXS: fragile X syndrome
HOPs: homotypic fusion and protein sorting
IDR: intrinsically disordered region
LLPS: liquid‐liquid phase separation
LRRK2: leucine rich repeat kinase 2
LSD: lysosomal storage disorder
LTD: long‐term depression
MPS‐IIIa: mucopolysaccharidosis type IIIa
NMJ: neuromuscular junction
PD: Parkinson's disease
RBP: RNA binding protein
RNP: RNA ribonucleoproteins
SMA: spinal muscular atrophy
SMN: survival motor neuron
SNARE: soluble *N*‐ethylmaleimide–sensitive factor attachment protein receptors
SOD‐1: super oxide dismutase 1
SV: synaptic vesicle
UTR: untranslated regions

## 1 Introduction

Throughout life, the nervous system constantly stores, retrieves, and processes information by coordinating structural changes in neuronal circuits with long‐term alterations in gene expression within individual neurons. These adaptive mechanisms support learning, memory, and behaviour, requiring precise regulation across various spatial and temporal levels. Neurons are highly specialised and structurally intricate cells designed for quick and accurate information processing. As post‐mitotic and long‐lived cells, they depend on robust mechanisms to maintain cellular integrity while adapting to evolving functional needs.

Neurons are characterised by their strong polarity, maintained by a complex cytoskeletal structure that creates distinct functional regions, including dendrites, the soma, and the axon. These regions can span large distances within the brain and body, posing unique logistical challenges compared to other cell types. Neuronal signalling relies heavily on precisely controlled, localised biochemical processes that occur outside the nucleus and at the main protein‐synthesis sites. Supporting these processes demands significant energy and calls for effective methods to distribute and regulate cellular resources. Because dendrites can extend hundreds of microns and axons up to a meter from the neuronal soma, maintaining a timely and localised protein supply to these compartments presents a significant logistical challenge. To overcome this, neurons traffic specific mRNAs to distal compartments and engage in on‐demand local protein synthesis, allowing for rapid, compartmentalised responses to changing demand. Remarkably, over 4800 mRNAs have been identified in distal neuronal processes (Glock et al. 2021), revealing an expansive local translatome capable of supporting diverse and changing neuronal states.

Maintaining proteostasis in neurons requires close coordination between protein synthesis and degradation (Ding et al. 2007). Proteostasis involves the precise balance of protein production and breakdown, which is crucial for cellular health. In neurons, which are long‐lived cells, this quality control system is vital for preserving function and avoiding the buildup of damaged or misfolded proteins.

Disruption of these precisely balanced regulatory systems can significantly impact neuronal function. Growing evidence suggests that problems with local protein homeostasis at synapses play a role in many neurodevelopmental and neurodegenerative disorders. Therefore, understanding how neurons maintain molecular and functional stability at distant synaptic sites is crucial for understanding normal nervous system function and the mechanisms underlying neurological diseases.

## 2 Proteostasis‐the Equilibrium Between Protein Synthesis and Degradation

As new proteins are produced, cells are continuously checking their quality, marking misfolded, damaged, or short‐lived proteins for destruction. The ubiquitin–proteasome system manages the rapid, selective degradation of specific proteins, while autophagy breaks down bulk cytoplasmic content and protein aggregates (Pohl and Dikic 2019), and even the ribosome itself (Kazibwe et al. 2019) (existing evidence for ribophagy has come from non‐neuronal cells). Both sides of the equation, synthesis and degradation, feedback on each other: when proteasomal or autophagic degradation is inhibited, it suppresses protein synthesis; and the same coordinated inhibition of degradation occurs when protein synthesis is inhibited. Together, this high level of coordinated protein synthesis and degradation allows neurons to rapidly remodel their proteome in response to changing developmental and activity needs. Dysfunction in neuronal proteostasis, at either the protein production or protein degradation levels, is common across multiple neurodevelopmental and neurodegenerative conditions.

## 3 Proteostasis at the Synapse

The synapse, the direct link between neurons and other target cells, is the smallest known functional unit of a neuron. Neurons can form thousands of synapses, which serve as postsynaptic sites that receive signals from other neurons and presynaptic sites that transmit signals, often located at considerable distances from the soma. This separation poses a significant challenge for maintaining synaptic integrity and function, particularly with respect to proteostatic control. Synaptic effectiveness relies on tightly regulating key cellular processes such as protein synthesis and the removal of old, unneeded, damaged or misfolded proteins. Instead of depending solely on long‐distance transport of proteins from the cell body to the synapse and vice versa, neurons can locally produce and break down proteins at the synapse itself. Ribosomes (Steward and Falk 1986), translation factors (Mendoza et al. 2021), and mRNAs are found in both presynaptic boutons and dendritic spines (Hafner et al. 2019); likewise, components of protein degradation, including the ubiquitin–proteasome system (Bingol and Schuman 2006) and the autophagy–endolysosomal pathway (Goo et al. 2017), are strategically located at synapses. These specialised pathways enable rapid, activity‐dependent changes in the synaptic proteome, which are essential for synaptic plasticity. In this review, we will outline several outstanding issues in the field of synaptic proteostasis, with a focus on synaptic protein synthesis and the autophagy‐endolysosomal pathway, and how neurological disease is linked to dysfunction in these pathways (Figure 1).

**FIGURE 1:**
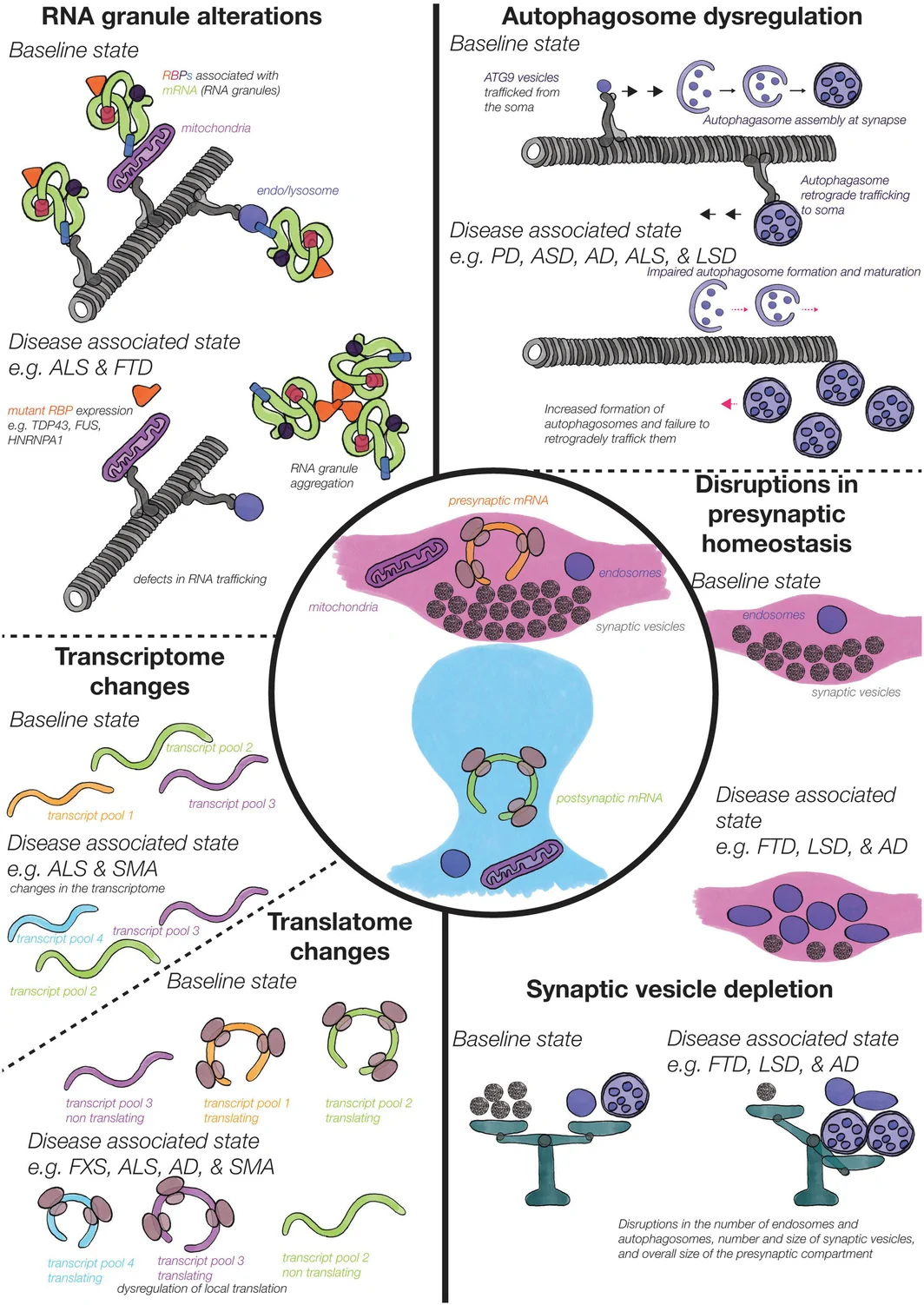
Common alterations in proteostasis are seen at the synapse in neurodegenerative and neurodevelopmental diseases. The diagram illustrates dysfunctions at various stages of local protein synthesis (left) and degradation (right), along with associated diseases for each change. It shows the presynaptic terminal (pink) and postsynaptic compartment (blue) at the center. Each panel compares normal states with disease conditions. RNA granule alterations: Normally, RBPs bind with mRNA in granules transported along microtubules, either directly or by hitchhiking on mitochondria and endo/lysosomes. In diseases such as ALS and FTD, mutant RBPs like TDP‐43 and FUS form larger, more viscous granules of altered composition that aggregate, leading to reduced RNA transport in axons (ALS, FXS). Transcriptome changes: The pool of localised transcripts is altered (ALS, SMA). Translatome changes: The active translating transcripts are altered, disrupting local protein synthesis (FXS, ALS, SMA). Autophagosome alterations: Normally, ATG9 vesicles are transported to the synapse, and autophagosomes form distally, maturing during dynein‐dependent retrograde transport to the soma. In disease, autophagosome formation or maturation is impaired (PD, ASD), and transport disruptions lead to accumulation in terminals (AD, ALS, LSD). Disruption in presynaptic homeostasis: Presynaptic endosomes increase (FTD, LSD, AD), synaptic vesicle numbers decrease (AD, LSD), and vesicles enlarge while presynaptic terminals shrink (AD). In disease conditions, the balance shifts toward degradative compartments, affecting synaptic vesicle and endosomal/autophagosomal systems. AD, Alzheimer's disease; ALS, amyotrophic lateral sclerosis; ASD, autism spectrum disorders; FTD, frontotemporal dementia; FXS, Fragile X syndrome; LSD, lysosomal storage disorder; PD, Parkinson's disease; RBP, RNA binding protein; SMA, spinal muscular atrophy.

## 4 Local Regulation of Protein Synthesis at Synapses

### 4.1 Resolving the Localisation and Dynamics of mRNA and Protein Synthesis Within Neurons

At the synapse, local regulation of protein synthesis is a critical component in sculpting and modifying the synaptic proteome. Local protein translation is a ubiquitous feature of the synapse, and the rate of local translation alters in response to synaptic activity.

Local protein production is widespread throughout the neuron, with both mature presynaptic terminals and dendritic spines routinely synthesising proteins even in the absence of exogenous stimulation (Hafner et al. 2019). Super‐resolution imaging at the single‐molecule scale has revealed that ribosomes are present at 85% of synapses, with 2 translation sites detected per synapse (Sun et al. 2021). Synapses significantly increase the amount of locally synthesised proteins in response to synaptic stimulation, although between neighbouring synapses, there is significant heterogeneity in the response levels (Sun et al. 2021). Significantly, mechanistically distinct forms of plasticity can modulate local protein synthesis, both pre‐ and postsynaptically (Hafner et al. 2019).

The diversity of RNAs which can be localised (Glock et al. 2021), and the apparent compartmentalisation of translation at synapses present a unique logistical challenge for a neuron. Namely, the relatively small size of an individual synapse (0.1‐1 μm^3^) and the large size of an individual molecule of mRNA. Take, for example, *Camk2a* mRNA, one of the most highly localised mRNAs in both excitatory axons and dendrites. CAMK2A protein is 478 amino acids in length, with a mass of 54 kDa. Its mRNA can be up to 4.9 kb in length, depending on 3′ untranslated region (UTR) usage, giving a mass of around 1.55 MDa, meaning that each *Camk2a* mRNA molecule is over 30 times the mass of its encoded protein. When stretched out, *Camk2a* mRNA would be over 2.7 μm in length, compared to around 172 nm for its protein. While compacting an RNA can reduce its overall size, large segments still need to be accessible for translation, meaning it would be impossible to package an RNA in a unit size comparable to its encoded protein. All mRNAs and proteins will show such size discrepancies, meaning that there simply is not enough available space in each synapse to host more than a handful of mRNAs at any given moment, given the experimental evidence for how densely compacted synaptic compartments are (Wilhelm et al. 2014). While the observation of monosome preference for translation in distal neuronal compartments (Biever et al. 2020), rather than traditional polysomal translation, does reduce added bulk, this alone cannot solve the logistical problem. Therefore, how does a neuron balance the need to make a diverse landscape of proteins from relatively large and bulky molecules of mRNA?

### 4.2 RNA Transport

mRNAs are not static molecules in cells; they can be directly trafficked via active transport along the cytoskeleton in both axons and dendrites. Beyond simply delivering mRNAs to distal compartments, RNA trafficking also addresses the logistical challenge posed by RNA size relative to the limited free space within an individual synapse. Building on early work, it was proposed that activity‐dependent recruitment of mRNA to synapses would be a vital aspect of RNA targeting (Doyle and Kiebler 2011), a phenomenon observed for an increasing number of mRNAs (Bauer et al. 2019; Donlin‐Asp et al. 2021; Grinman et al. 2021; Yoon et al. 2016). This mechanism, described as sushi‐belt recruitment of mRNA, posits that mRNAs are dynamically recruited as needed to serve as templates for plasticity‐specific translation. How specific mRNAs would be identified and recruited to synapses undergoing plasticity has never been explained, however. Recent work challenges the specificity and selectivity of this model, finding that by tracking multiple mRNA targets in live cells and visualising the translation of endogenous proteins in real‐time, it revealed a widespread alteration in dendritic mRNA dynamics during distinct forms of synaptic plasticity, regardless of whether the protein was being made (Donlin‐Asp et al. 2021). This suggests that RNAs may be transiently and non‐specifically recruited to synapses undergoing plasticity to rapidly diversify the mRNAs available for the production of specific proteins.

The trafficking of mRNA within a cell requires both *cis*‐elements, which are intrinsic to the RNA molecule, and *trans*‐elements, such as RNA‐binding proteins. To achieve this, RNA‐binding proteins (RBPs) bind either the coding sequence of the transcript or its 5′ or 3′ UTRs, forming complexes known as RNA ribonucleoproteins (RNPs). RNPs are then transported to the intracellular location of interest by direct or indirect interaction with motor proteins. Importantly, mRNAs are also regulated during transport: RBPs within transport RNPs can keep transcripts translationally repressed until they arrive at their target or encounter the right stimulus. This means that the location of an mRNA and the timing of its translation are controlled separately (Krichevsky and Kosik 2001).

#### 4.2.1 The Classical “Zipcode” View

Early research on the first identified subcellularly localised *β‐actin* revealed a specific 54‐nucleotide segment, known as the zipcode, essential for its localisation (Kislauskis et al. 1994). This segment became the model for understanding how RNAs are localised within cells, featuring a short consensus sequence that serves as a binding site for trans‐acting RBPs. These RBPs act as mediators, connecting RNA to the motor‐based cytoskeletal transport system to direct it to its intracellular destination (Ross et al. 1997; Zhang et al. 2001).

Although the model is attractive and approachable, the successful identification of definitive zipcodes in other RNAs over the past few decades has been limited. Some putative zipcodes have been identified (Ciolli Mattioli et al. 2019; Huang et al. 2003; Kobayashi et al. 2005; Oe and Yoneda 2010; Rook et al. 2000), but most are only loosely described regions within the 3′UTR, especially when compared to the defined sequence element in *β‐actin*. With the rise of omics‐based profiling of local transcriptomes and the expansion from tens to thousands of known localised transcripts (Cajigas et al. 2012; Davis et al. 1987; Eberwine et al. 2001; Garner et al. 1988; Glock et al. 2021; Middleton et al. 2019; Perez et al. 2021), there is still a significant lack of consensus sequences that could serve as functional zipcodes. One explanation is that, unlike the sequence‐specific *β‐actin* zipcode, many zipcodes might be RNA secondary‐ or tertiary‐structural elements. In fact, even the beta‐actin zipcode involves both a sequence component and a flexible loop that facilitates interactions with domains in ZBP1 (Chao et al. 2010). Another emerging possibility is that, instead of relying on the traditional zipcode model, alternative mechanisms—such as regulation of RNA stability and organelle hitchhiking—might direct RNA localisation.

#### 4.2.2 RNA Stability

Alternative splicing is another means to direct the destination of an mRNA, with alternative last exons often conferring intracellular localisation. Gene‐distal alternative last‐exon isoforms are four times more likely to be localised to neurites than gene‐proximal isoforms (Taliaferro et al. 2016). Undisputed in the functional localisation of RNAs is the role of the 3′UTRs. 3′UTRs are significantly longer in the mammalian brain than in other cell types (Shin and Brangwynne 2017; Tushev et al. 2018), and the use of alternative 3′UTR isoforms, defined by alternative polyA selection sites, has been identified as a novel mechanism to direct differential localisation of functionally diverse alternative protein isoforms. By separating soma from neurites, approximately 600 genes with differentially localised 3′UTRs have been identified; alternative 3′UTRs were found to direct different transcript isoforms from the same gene to localise at distinct subcellular compartments (Ciolli Mattioli et al. 2019).

The idea behind these longer 3′ UTRs is that they would provide increased regulatory information for neuronal transcripts. A study that tested the localisation‐regulatory capacity of thousands of sequence fragments from endogenous mouse 3′UTRs identified regulatory activity peaks in several 3′UTRs. Sequences derived from these peaks were both necessary and sufficient for RNA localisation to neurites in mouse and human neuronal cells (Arora et al. 2022). Interestingly, the identified localisation elements ranged from tens to hundreds of nucleotides, indicating significant sequence complexity in cis‐regulatory elements, consistent with a complex balance of factors required for transcript localisation, including RNA stability. Indeed, localised RNA transcripts are, on average, longer‐lived than somatically enriched RNAs (Tushev et al. 2018). This suggests that regulation of RNA stability may be an essential component for localising RNAs in distal neuronal compartments. However, the exact nature of this relationship is still unclear: do localised mRNAs tend to be more stable because of their localisation—perhaps protected by transport RBPs and RNP components they bind to—or because they are inherently more stable? Clarifying this will be key to determining whether RNA stability drives localisation or results from it.

Notably, in an attempt to identify neuronal zipcodes, an identification protocol (N‐zip) using massively parallel reporter assay identified the let‐7 binding site and (AU)_n_ motif as de novo “zipcodes” (Mendonsa et al. 2023; Nicastro et al. 2017). While termed ‘zipcodes’, these sequences regulate RNA stability and are consistent with a model in which RNA stability regulation is a key determinant of RNA localisation.

The issue with RNA stabilisation is that while it would prolong an RNA molecule's lifespan, it alone cannot drive RNA transport. And while passive diffusion of RNAs in the cytosol can enrich them over tens of microns (Katz et al. 2016; Wu et al. 2012), in neurons, such diffusive movement is only really seen for localised mRNAs during translation (Donlin‐Asp et al. 2021; Turner‐Bridger et al. 2018) and would be too slow to explain localisation over hundreds of microns on the timescale of an mRNA half‐life.

#### 4.2.3 RNA Transport Granules‐Or RNPs?

Traditionally, the subcellular localisation of mRNAs in neurons is understood to occur through the direct transport of RNP complexes along cytoskeletal tracks, which is essential for localised gene expression in axons and dendrites. In this model, mRNAs are bound to RBPs within transport granules that interact with molecular motors, enabling long‐distance, bidirectional movement along microtubules and short‐distance positioning along actin filaments (Hirokawa et al. 2010; Holt and Bullock 2009). Microtubule‐based motor proteins, especially kinesins and dynein, drive the antero‐ and retrograde transport within axons and dendrites, while myosin motors manage localised movements and anchoring near synapses via actin (Buxbaum et al. 2015; Kanai et al. 2004). Live‐cell imaging demonstrates that these granules exhibit saltatory motion and frequently change direction, highlighting their dynamic interactions with various motor complexes, influenced by neuronal activity and signalling pathways (Donlin‐Asp et al. 2021; Dynes and Steward 2007; Knowles et al. 1996; Turner‐Bridger et al. 2018; Yoon et al. 2016). Unlike larger, well‐characterised RNA granules such as p‐bodies and stress granules (Decker and Parker 2012), germ granules (Phillips and Updike 2022), and oskar granules (Bose et al. 2022), the precise nature and organisation of RNA transport granules remain poorly understood.

Liquid–liquid phase separation (LLPS), proposed for other RNA granules, mainly drives the formation of most RNP granules (Shin and Brangwynne 2017). It enables RBPs to create concentrated, membraneless RNA condensates or granules that compartmentalise RNAs within cells. Research on various RNA granules, such as stress granules and processing bodies (Decker and Parker 2012), shows that LLPS is driven by multivalent, weak interactions between intrinsically disordered protein regions and RNA (Franzmann and Alberti 2019; Gomes and Shorter 2019). These interactions act as scaffolds, promoting cooperative binding and assembly. The resulting condensates are dynamic and liquid‐like, allowing rapid exchange of RBPs and RNA. Factors like RNA concentration (Bauer et al. 2022), post‐translational modifications of RBPs (Hofweber and Dormann 2019), or cellular stress can influence condensate formation and dissolution (Yoo et al. 2019). Despite extensive evidence from in vitro and overexpression studies, the importance of LLPS within intact cells at physiological protein and RNA levels remains unclear (Hedtfeld et al. 2024). RNA transport granules are proposed to include various co‐regulated RNAs, translational repressors, and ribosomes to facilitate the delivery of mRNA and ribosomes within the cell (Bauer et al. 2023). Neuronal depolarization reorganises these granules, making ribosomes less compact and initiating local translation (Krichevsky and Kosik 2001). Single‐molecule imaging of β‐actin transcripts showed that RNA granules are densely packed, but neuronal stimulation renders mRNA and ribosomes accessible (Buxbaum et al. 2014). Although studies of the RNA granule interactome reveal ribosomes and repressors, indicating multiple subtypes (Kipper et al. 2022), the exact RNA copy number in these granules remains uncertain; most evidence favours single‐copy transcripts with minimal overlap with others (Batish et al. 2012; Donlin‐Asp et al. 2021; Mikl et al. 2011; Yoon et al. 2016). This ambiguity may stem from a lack of clarity in terminology, as describing these RNAs as packaged in granules implies a higher‐order organisation, such as seen with stress granules (Jain et al. 2016). More accurately, a single RNA bound to various proteins should be called a RNP complex, which better reflects the transport state of RNAs.

Puzzling is how transporting single RNAs fits into the overall argument for local protein synthesis—namely, it provides a rapid and efficient way to adapt the local proteome faster and more precisely than protein trafficking alone—along with the sheer numbers of mRNAs which can be localised. It raises a serious question, even with RNA capture at synapses: how can neurons produce enough copies of hundreds to thousands of different transcripts to provide sufficient resources to make any noticeable impact on the proteome, if each of these is being trafficked one at a time?

These two arguments represent contrasting points, and we consider this tension an open question in the field. One side suggests that because of mRNA size and synaptic compartment density (Wilhelm et al. 2014), only a limited number of mRNAs can fit in a synapse at once, setting a ceiling on local mRNA quantity. Conversely, if mRNAs are transported individually, supplying a wide variety of transcripts in significant numbers to influence the local proteome would require many separate delivery events. An often‐overlooked point that might reconcile these views is that translation does not destroy the mRNA: a single mRNA can be translated multiple times, producing many proteins during its lifespan. Thus, the number of mRNA copies needed at a synapse depends not only on the number of proteins required but also on how often each mRNA is translated and how long it remains active. Interestingly, localised mRNAs tend to last longer than those in the soma (Tushev et al. 2018), which boosts protein production per transcript. Additionally, the preference for monosome over polysome translation in distant compartments (Biever et al. 2020) might temporarily limit each mRNA's output while reducing its physical space. It remains unclear whether individual mRNAs are repeatedly translated at a single synapse or shared among neighbouring synapses through dynamic recruitment (Donlin‐Asp et al. 2021). To clarify these issues, precise measurements of mRNA copies, translation rates, and protein yields at individual synapses are essential.

### 4.3 Organelle Hitchhiking–An Efficient Method to Transport RNAs to Distal Compartments?

One possible approach to ensuring that neurons deliver sufficient RNA via single RNPs is to hijack organelle transport. Neuronal RNA trafficking is increasingly understood to involve the endolysosomal system, which acts as both a mobile platform for RNA transport and a regulator of localised translation. Early fungal studies showed that mRNAs associate with motile endosomes for long‐distance movement, a mechanism later proven to be conserved in neurons (Baumann et al. 2014; Cioni et al. 2019). In neurons, Rab7‐positive late endosomes cotraffic with mRNAs, ribosomes, and translation machinery along axons, supporting localised protein synthesis often near mitochondria (Cioni et al. 2019; Liao et al. 2019). Lysosomes also play a role by tethering RNA to their membranes through annexin A11 (ANXA11), facilitating continuous axonal transport of RNA cargo. Mutations in ANXA11 linked to amyotrophic lateral sclerosis (ALS) disrupt this interaction, impairing RNA localisation (Liao et al. 2019).

In addition to the endolysosomal system, mitochondria associate with subsets of nuclear‐encoded mitochondrial mRNAs at the outer mitochondrial membrane, facilitating cotranslational import and local regulation of metabolic capacity (Fazal et al. 2019; Lesnik et al. 2014). Apart from mitochondrial protein transcripts, some evidence suggests that cytosolic RNAs also associate with and potentially traffic with mitochondria (Murali Mahadevan et al. 2026). Collectively, these observations support a model in which RNA localisation in neurons could be achieved through coordinated interactions with multiple organelles, in addition to the classic direct motor‐based transport. Intriguingly, this may also solve the targeting of RNAs to distinct subcellular regions of a neuron issue as well, as the neuron has robust and developed pathways for cargo sorting to the axon and dendrite, and by integrating RNA transport with these intracellular trafficking pathways, it would present an efficient method for regulating spatially restricted gene expression.

## 5 Synaptic Protein Degradation

The intracellular degradation of proteins is also essential for cellular homeostasis. Protein degradation at synapses occurs mainly through two interconnected pathways: the ubiquitin–proteasome system (UPS) and the autophagy–lysosomal pathway (Pohl and Dikic 2019). In the UPS, ubiquitin‐tagged proteins are directed to the proteasome for breakdown. Proteasomes are present in both presynaptic terminals and dendritic spines, where they support activity‐dependent synaptic proteome remodelling: neuronal activity prompts proteasome recruitment into dendritic spines (Bingol and Schuman 2006). The UPS also influences the postsynaptic density composition (Ehlers 2003) and acutely modulates presynaptic proteins like the vesicle‐priming protein DUNC‐13, affecting neurotransmitter release (Speese et al. 2003). However, for this review, we will focus on macroautophagy and the endolysosomal system, as the literature provides the strongest evidence of their importance in synaptic pathophysiology. Autophagy (or “self‐eating”) involves the delivery of cargo to a nascent organelle (the autophagosome), which forms following elongation of intracellular membranes. Autophagosomes then fuse with endolysosomes, which deliver additional cargoes and degradative enzymes necessary for the degradation of organellar cargo. This process proceeds in a highly controlled fashion using a well‐described intracellular signalling cascade. Indicating the importance of this process at distal neuritic locations, loss of autophagy can cause defects in axon outgrowth and guidance (Ban et al. 2013), dendritic branching and maturation (Clark et al. 2018), synapse formation (Hassan and Hiesinger 2023), synaptic activity (Yang et al. 2022) and synapse pruning (Tang et al. 2014). Furthermore, mutations in key autophagy and endolysosomal‐associated proteins have been identified as genetic causes of both neurodevelopmental and neurodegenerative conditions.

Autophagy‐related genes (ATG) are highly conserved across species. In the canonical autophagy pathway, protein degradation follows the following steps: initiation, phagophore formation and elongation, autophagosome completion, fusion with the endolysosome to create an autolysosome, and finally degradation and recycling within the autolysosome. For comprehensive reviews on autophagy initiation and progression, see (Aman et al. 2021; Dikic and Elazar 2018). In brief, we will describe a few key players here which are necessary to reference when describing the role of autophagy in disease. The activation and translocation of the ULK1 complex is the first step in the autophagy process, followed by the generation of phosphatidylinositol‐3 phosphate (PI3P) (Grasso et al. 2018). WIPI proteins are PI3P‐binding proteins which then recruit downstream ATG proteins (Grimmel et al. 2015). The membrane deposition of ATG16L1 recruits membrane localisation of the ATG‐12‐ATG‐5 complex and LC3, which promotes the membrane expansion of the phagophore (Fujita et al. 2008; Mizushima et al. 1998). ATG9 vesicles deliver lipids and machinery to the growing phagophore (Holzer et al. 2024). Autophagosome‐lysosome fusion is mediated by a complex machinery, involving tethering proteins such as the Homotypic fusion and protein sorting (HOPs) complex, soluble *N*‐ethylmaleimide–sensitive factor attachment protein receptors (SNAREs), rab proteins and ATG8. The HOPs complex resides on the lysosome and interacts with the autophagosome‐localised SNARE syntaxin 17 to mediate fusion between the two organelles (Jiang et al. 2014).

### 5.1 Synaptic Macroautophagy

Classically, studies on the molecular mechanisms of autophagy have been conducted on immortalised cell lines. However, as discussed for local protein translation, neurons also employ specialised machinery for degradation in the distal synaptic regions of their neurites. Neuronal autophagy is essential for synaptic function (for a review, see (Karpova et al. 2025)), and compartmentalised synaptic autophagy is a product of crosstalk between multiple membrane trafficking pathways. At the axo‐synaptic terminal, this hybridised form of local protein degradation combines classical features of autophagy with specialised input from molecular motors for retrograde trafficking and moonlighting synaptic vesicle (SV) trafficking proteins. Profiling of autophagic vesicles from mouse brain has revealed that a quarter of the enriched autophagic content is composed of synaptic proteins, highlighting the importance of autophagy in the maintenance of synaptic architecture and function (Kallergi et al. 2023).

#### 5.1.1 Presynaptic Autophagosome Biogenesis

Neuritic autophagosomes initiate distally and mature as they traffic back toward the cell body (Maday et al. 2012) in a dynein‐dependent fashion (Cheng et al. 2015). Although autophagy operates at a basal level at distal axo‐synaptic locations, as can be seen in unstimulated neurons in culture (Maday et al. 2012), there are also numerous stimuli known to trigger presynaptic autophagy. The sonic hedgehog signalling pathway, for example, enhances synaptic autophagy; EM of cultured hippocampal neurons exposed to sonic hedgehog show numerous autophagosomes in synaptic terminals (Petralia et al. 2013). Another trigger for synaptic autophagosome biogenesis is neuronal activity, with increased synaptic activity significantly upregulating autophagic trafficking from the synapse to the cell body (Wang et al. 2015). Not only does increased synaptic activity upregulate retrograde autophagic trafficking, autophagy itself can also modulate presynaptic function. Treatment with the mTOR inhibitor rapamycin induced the formation of bouton‐adjacent axonal autophagosomes, with a concomitant decrease in the number of SVs at presynaptic terminals, demonstrating a direct effect of increased autophagy on reduced synaptic release (Hernandez et al. 2012). While the molecular mechanisms governing this process are not fully understood, there is significant interplay between proteins involved in SV trafficking and in presynaptic autophagosome initiation and trafficking.

#### 5.1.2 Interplay Between Synaptic Vesicle Proteins and Autophagy Markers

There is a clear balance presynaptically between SV number and autophagosome biogenesis, indicating significant crosstalk between these two processes. Presynaptic autophagy is coupled to the SV cycle via ATG‐9 (Yang et al. 2022) with the SV kinesin UNC‐104/KIF1A transporting ATG‐9 to presynaptic sites (Stavoe et al. 2016). Additionally, the presynaptic active zone protein bassoon plays a key role in presynaptic autophagy, with gain‐ or loss‐of‐function mutants respectively suppressing or enhancing it. Bassoon fulfils this role via interaction with the autophagy protein atg5 (Okerlund et al. 2018). Interestingly, conditional knockout of atg5 and the resulting loss of neuronal autophagy leads to axonal accumulation of tubular endoplasmic reticulum and increased neurotransmission, indicating that neuronal autophagy is essential for regulating neurotransmission (Kuijpers et al. 2022).

##### 5.1.2.1 Rabs, SV Trafficking and Autophagy

Rab proteins are crucial molecular switches that control intracellular trafficking. A synaptic‐specific role for Rab26 proteins and their GEFs in linking SVs and autophagy has been reported. Overexpression of Rab26 leads to clusters of SVs, which were observed to co‐localise with several markers of the autophagy pathway (Atg16L1, LC3B and Rab33B) (Binotti et al. 2015). Furthermore, Pleckstrin homology containing family member 5 (Plekhg5) was identified as a GEF for Rab26, and when this Rab26 GEF was knocked out in cultured motor neurons, the axons showed a reduction in the number of autophagosomes (Lüningschrör et al. 2017).

While Rab26 mediates its effects linking SVs to autophagy through physical co‐localisation at the presynaptic terminal, recent work has identified a Rab that can influence synaptic autophagy despite being localised to the soma. In a large Drosophila screen designed to identify soma‐localised modifiers of presynaptic autophagy, Rab39 was identified as a synaptic autophagy regulator, with active Rab39 found to selectively control delivery of autophagy‐related Atg9‐positive vesicles to synapses (Kilic et al. 2025). This shows a role for cross‐compartment control of autophagy mechanisms, from the soma to the presynaptic terminal.

## 6 Neuronal Proteostasis and Disease

### 6.1 RBPs and Associated Proteins in Neurodegenerative and Neurodevelopmental Disease

With thousands of mRNAs localised and translated in neuronal processes, understanding how the brain controls where mRNAs localise and are translated, and the significance of their regulated translation are critical outstanding questions in the field. Multiple RBPs have been implicated in the pathogenesis of both neurodevelopmental and neurodegenerative diseases. In particular, mutations within or aggregation of RBPs are a widespread and conserved feature of ALS, regardless of the gene mutated.

ALS is a neurodegenerative disease characterised by the degeneration and death of motor neurons. Mutations in the intrinsically disordered regions (IDRs) of multiple RBPs are frequently associated with familial ALS, and RBPs are additionally often found to be mislocalised and/or aggregated in both sporadic and familial ALS (Taylor et al. 2016). ALS primarily affects both the upper and lower motor neurons. This indicates that the physiological role of RBPs (splicing, granule formation, granule trafficking, translation repression and local translation) is a key player in disease, and a better understanding of RBPs in these processes is essential to furthering our understanding of disease.

TDP‐43 is a crucial RNA/DNA‐binding protein that normally resides in the nucleus, influencing RNA metabolism by regulating splicing and stability. Importantly, TDP‐43 dysfunction is a hallmark of multiple neurodegenerative diseases. Under disease conditions, TDP‐43 mislocalizes to the cytoplasm, where it becomes ubiquitinated and forms aggregates, leading to neuronal dysfunction and death (Jo et al. 2020).

TDP‐43 forms RNPs that undergo bidirectional transport in neurons, which facilitates the microtubule‐dependent delivery of mRNA to distal neuritic locations. ALS‐linked mutations in TDP‐43 increase the size of dendritic RNA granules (Liu‐Yesucevitz et al. 2014), and impair the axonal transport of TDP‐43 positive RNPs (Alami et al. 2014). TDP‐43 RNP granules in axons display liquid‐like properties, and ALS‐linked mutations increase the viscosity of these granules and disrupt transport dynamics (Gopal et al. 2017). Loss of TDP‐43 altered the transcriptome of the axonal compartment and also caused reduced protein synthesis in the axon (Briese et al. 2020). Axonal accumulation of TDP‐43 promotes G3BP‐1 positive RNP assemblies, and inhibits local protein synthesis in the distal axon (Altman et al. 2021; Briese et al. 2020). Thus, evidence exists that in TDP‐43‐associated ALS, multiple steps of local translation are altered, from granule formation, through transport to distal protein synthesis.

Another RBP which plays a role in ALS is FUS, mutations in which account for 4% of familial ALS cases (Volk et al. 2018). ALS‐mutant FUS has the ability to sequester wildtype FUS within stress granules (Vance et al. 2013), and ALS‐associated FUS mutations reshape the RNA and protein composition of stress granules. The expression of mislocalised ALS mutant FUS is sufficient to impact the dynamics of stress granules and to strongly modify their transcriptome by enriching AU‐rich RNAs at the expense of GC‐rich transcripts (Mariani et al. 2024), again showing the effect of ALS‐associated mutation in FUS impacts multiple steps in proteostasis. However, how this specifically impacts the synaptic transcriptome is not yet known, although FUS itself is found presynaptically (Schoen et al. 2015).

The impact on local proteostasis is also evident in other forms of neurodegenerative disease. An expanded polyglutamine tract in *ATXN2* (Ataxin‐2) can cause spinocerebellar ataxia Type 2, which primarily affects the cerebellum. Interestingly, intermediate repeat expansions are a significant risk factor for ALS (Elden et al. 2010). RNP granule assembly via the IDR of Ataxin 2 is required for translation‐dependent long‐term memory (Bakthavachalu et al. 2018), indicating a synaptic‐specific role for this protein and inferring synaptopathy in expanded variants of the gene.

Clear evidence for the importance of RNA granule transport comes from additional genetic models of ALS. Annexin A11, identified as the linker protein attaching hitchhiking RNA granules for transport, is mutated in ALS/FTD. ALS causative mutations disrupt the RNA granule binding to annexin A11, thus impairing the lysosome‐mediated transport of these granules, which is necessary for local axonal protein translation (Liao et al. 2019).

The importance of correct splicing mediated by RBPs is also prevalent in the literature. In zebrafish, the loss of SFPQ (an RBP and splicing factor linked to ALS) in neurons produces aberrant intron‐retaining transcripts coding for neuron‐specific proteins that accumulate in neurites. Some of the identified aberrant intron‐retaining transcripts are prematurely terminated within the retained intron. Within the retained intron, the mRNAs undergo intronic polyadenylation, nuclear export, and localise to neurites both in vitro and in vivo, with the same hallmark seen in familial and sporadic ALS tissue RNA (R. Taylor et al. 2022). SFPQ also operates in sensory neuron axons, where it binds and co‐assembles multiple mRNAs within axonal transport granules. It is essential for the axonal localization of this RNA regulon, which includes Lmnb2 and Bclw mRNAs, and for neurotrophin‐dependent axon survival (Cosker et al. 2016). This pathway also connects RBP function to axonopathy beyond traditional neurodegenerative diseases: the chemotherapeutic drug paclitaxel disrupts the transport of RNA granules containing bclw mRNA and SFPQ, decreasing local Bclw translation and causing IP3R1‐dependent axon degeneration in chemotherapy‐induced peripheral neuropathy (Pease‐Raissi et al. 2017).

While ample evidence exists for the importance of RNA processing, localisation and translation in neurodegenerative disease, mutations in RBPs and proteins associated with RBPs have additionally often been associated with neurodevelopmental disorders. Strong evidence is seen in the case of fragile X syndrome (FXS). Mutations in the *FMR1* gene, which codes for fragile X messenger ribonucleoprotein (FMRP), cause FXS, the most common form of inherited intellectual disability. FMRP promotes RNA localisation to neuronal projections through interactions between its RGG domain and G‐quadruplex RNA sequences (Goering et al. 2020). FMRP is an RBP, and neurons lacking FMRP exhibit altered RNA transport and protein translation in dendritic spines (Kao et al. 2010). Presynaptic FMRP is a critical regulator of presynaptic plasticity, as deletion of presynaptic FMRP blocks structural and functional plasticity of glutamatergic axon terminals (Monday et al. 2022).

Evidence for the importance of chaperones in translation fidelity is evident in the case of spinal muscular atrophy (SMA). Loss‐of‐function mutations in the survival motor neuron (SMN) protein cause SMA, a rare developmental disorder characterised by progressive muscle weakness due to loss of motor neuron function (Lefebvre et al. 1995). SMN is part of a large macromolecule complex that acts as a molecular chaperone for spliceosomal snRNP assembly (Pellizzoni et al. 2002) and mRNPs (Donlin‐Asp et al. 2017), and forms granules that traffic bidirectionally in neurites (Zhang Honglai et al. 2003). Loss of SMN results in significant transcriptomic alterations in axons, with transcripts associated with axon growth and synaptic activity downregulated (Saal et al. 2014). Interestingly, SMN associates with polyribosomes and can repress translation (Sanchez et al. 2013), potentially either through direct association with the ribosomes or via its well‐known association with RBPs (Donlin‐Asp et al. 2017; Fallini et al. 2016, 2011). Conversely, SMN primed ribosomes have been found to modulate translation of SMA‐associated transcripts, in this case acting as a translation activator (Lauria et al. 2020).

These studies show that controlling RNA metabolism, RNP granules, and local protein production is key to maintaining neuronal proteostasis. Neurodegenerative and neurodevelopmental disorders often result from disrupted processes like mRNA splicing, transport, localisation, translation regulation, and granule formation, rather than a single defect. Minor disruptions can greatly impact the local proteome, affecting synaptic function, plasticity, and neuronal survival, especially in neurons with long axons. Understanding RNA localisation and translation in healthy neurons is crucial for uncovering disease mechanisms and developing therapies to restore proteostasis.

### 6.2 Synaptic Dysregulation of Endolysosomal Trafficking and Autophagy Is Often Associated With Neurodegenerative Disease

Protein degradation pathways are essential for neuronal homeostasis, with defects in autophagy and endolysosomal degradation often associated with both neurodevelopmental and neurodegenerative conditions. Genetic models of familial forms of neurodegenerative disease have been essential for advancing our understanding of disease‐relevant molecular mechanisms. Frequently, models of genetic forms of neurodegenerative disease present with early synaptic pathology, and defects in vesicular trafficking pathways, such as protein degradation, are commonly reported across multiple forms of neurodegenerative disease. Thus, understanding the molecular mechanisms underlying early synaptic dysfunction is extremely important for identifying and designing novel therapeutic interventions.

#### 6.2.1 Parkinson's Disease

Parkinson's disease (PD) is characterised by the pathological accumulation of ɑ‐synuclein, which plays a physiological role in the SV cycle by promoting SNARE complex assembly (Burré et al. 2010). Multiple PD‐associated genetic mutations play a fundamental role in SV endocytosis, further underscoring the delicate link between SV turnover and synaptic autophagy, and PD causative mutations have been linked to defects in synaptic autophagy.

Mutations in synaptojanin have been identified as causal for early‐onset familial PD (Krebs et al. 2013). Synaptojanin is a presynaptic lipid phosphatase important for dephosphorylating phosphoinositides, a crucial step in SV endocytosis (Di Paolo and De Camilli 2006). Significantly, synaptojanin is required for autophagosome maturation at synapses; a PD mutation in synaptojanin blocks presynaptic autophagy and induces dopaminergic neuron loss in flies (Vanhauwaert et al. 2017). Interestingly, this role for synaptojanin in autophagosome maturation is separate from the role of synaptojanin in SV endocytosis, with SV turnover unaffected by knock‐in of mutant synaptojanin (Vanhauwaert et al. 2017).

Mutations in leucine rich repeat kinase 2 (LRRK2), which increase its kinase activity, are a common cause of PD. LRRK2 is a particularly interesting gene in the aetiology of neurodegenerative disease, as some mutations in PD are disease‐causative, while other mutations are not fully penetrant. Furthermore, elevated LRRK2 activity is frequently found in sporadic PD (without LRRK2 mutation), indicating that LRRK2 activity is strongly tied to disease development (Bailey and Cookson 2024). LRRK2 is a kinase which phosphorylates numerous proteins that are necessary for SV endocytosis, including auxilin, dynamins 1–3, synaptojanin‐1, and endophilinA (Islam et al. 2016; Matta et al. 2012; Nguyen and Krainc 2018; Stafa et al. 2014). Of note, LRRK2 phosphorylation of the endophilinA BAR domain promotes the formation of highly curved membranes that serve as docking sites for autophagic factors, and imbalances in endophilinA phosphorylation cause dopaminergic neuron degeneration (Soukup et al. 2016).

Interestingly, although many regulators of synaptic autophagy are themselves synaptically localised, synaptic autophagy can also be regulated in a cross‐compartmental fashion. Kilic et al. recently reported a soma‐centred mechanism which controls synaptic protein turnover by autophagy in a Drosophila model. Rab39, a genetic contributor to PD, is soma‐localised, yet loss or disease‐causative mutations of Rab39 upregulate presynaptic autophagy and lead to dopaminergic neuron loss (Kilic et al. 2025). Rab39 controls this process via Shot‐ and Unc104/KIF1A‐mediated synaptic delivery of Atg9‐positive vesicles.

Highly significantly, in addition to PD causative mutations resulting in presynaptic autophagy defects, the converse is also true. This is evident from knock out models of autophagy‐related proteins. Deletion of *Atg7* in the mouse brain results in presynaptic accumulation of ɑ‐synuclein and LRRK2 proteins, showing that alteration to autophagy itself causes presynaptic accumulation of PD‐relevant pathologies (Friedman et al. 2012).

#### 6.2.2 Alzheimer's Disease

Alzheimer's disease (AD) is the most common form of dementia, and cellular protein degradation defects have frequently been reported in AD. Several studies have also localised these protein degradation defects specifically to synaptic compartments; for example, immuno‐electron microscopy shows an abundance of autophagosomes and autolysosomes in neurites in AD patient brains (Nixon et al. 2005).

Mutations in presenilin 1 or 2 (PSEN1/PSEN2) cause a familial form of young‐onset AD, and mutations in the amyloid precursor protein (APP) also cause AD (Tanzi 2012). Ultrastructural analysis of the hippocampus of 4–6‐month‐old AD model mice bearing both PSEN1 and APP mutations revealed an accumulation of autophagosomal structures in the presynaptic terminals of plaque‐associated neurites (Sanchez‐Varo et al. 2012). When PSEN2 was either knocked out or a familial AD (FAD) associated PSEN2 mutant was expressed in an APP knock‐in mouse model, the neurons presented with endolysosomal deficits and smaller presynaptic compartments in the mossy fibre of the disease model hippocampus (Perdok et al. 2024). The number of SVs per presynaptic terminal was reduced in the disease model synapses, and the authors observed a number of enlarged SVs in the mutant synaptic terminals (Perdok et al. 2024). It's possible that these enlarged SVs may represent presynaptic endosomes, observed in the disease model due to PSEN2 KO or FAD mutant expression, although this currently would be difficult to show, as the field lacks a definitive marker for presynaptic endosomes/endolysosomal structures.

#### 6.2.3 Frontotemporal Dementia

Frontotemporal dementia (FTD) is caused by loss of neurons in the frontal and temporal lobes. Familial FTD‐causative mutations are often associated with endolysosomal dysfunction (Todd et al. 2023). SV trafficking defects are also frequently reported in models of both FTD and ALS (Clayton et al. 2024). However, direct evidence linking endolysosomal dysfunction and presynaptic function is harder to find. Recent studies on CHMP2B (part of the ESCRT‐III complex and a rare cause of FTD and ALS) have provided a more direct link. Neuritic endolysosomal trafficking was found to be significantly impaired in mouse CHMP2B‐FTD primary neurons (Clayton et al. 2018). EM of the synapses revealed an accumulation of endosomal structures in presynaptic terminals, and a cumulative downregulation of SV release following sustained synaptic stimulation (Clayton et al. 2022), suggesting interplay between the SV cycle and endolysosome biogenesis or resolution that is impaired in the presence of mutant ESCRT‐III complex.

#### 6.2.4 Amyotrophic Lateral Sclerosis

Mutations in genes associated with the autophagy and protein degradation pathway are often associated with ALS (*SOD1, VCP, OPTN, UBQLN2, SQSTM1*) (Taylor et al. 2016), however direct evidence of a synaptic deficit in synaptic protein degradation is not abundant in the literature. Some evidence of a synaptic localised pathophysiology is available‐ for example, in a mouse model of ALS expressing mutant superoxide dismutase 1 (SOD‐1), motor neurons were found to form large autophagosomal vacuoles early in disease progression. Interestingly, inhibiting autophagy by conditionally knocking out *Atg7* in the mutant SOD1 mouse motor neurons was found to exacerbate neuromuscular junction (NMJ) pathology (Rudnick et al. 2017).

### 6.3 Links Between SVs, Endosomes and Autophagosomes

Of note, many genes linked to familial neurodegenerative disease are important for SV turnover at the presynaptic terminal. As discussed above, the SV cycle is intrinsically and tightly linked to endolysosome and autophagy biogenesis and trafficking; thus, defects in SV turnover at the presynaptic terminal are likely to impact these complexly intertwined degradative pathways.

### 6.4 Chaperones at the Synapse

Molecular chaperones are important mediators of cellular homeostasis, through roles in assisting folding nascent proteins, refolding misfolded proteins and tagging proteins for degradation via the ubiquitin‐proteasome system or autophagy‐lysosomal pathway. Chaperones are critical quality control checkpoints which act to clear out defective or toxic protein assemblies, and failure in protein quality control is often seen in neurodegenerative disease (for a review, see Ciechanover and Kwon 2017). Several synaptic chaperones have important roles in neurotransmission, and are also heavily implicated in neurodegenerative disease (see (Gorenberg and Chandra 2017) for a comprehensive review on synaptic chaperones).

Cysteine string protein α (CSPα) is a dedicated chaperone that prevents the misfolding and aggregation of the SNARE protein SNAP‐25, and CSPα‐deficient mice exhibit neurodegenerative changes (Fernández‐Chacón et al. 2004). Mutations in CSPα cause adult neuronal ceroid lipofuscinosis, a rare lysosomal storage disorder (Benitez et al. 2011; Nosková et al. 2011). The synaptic function of chaperones has also been strongly linked to PD, with mutations in the synaptic chaperones Auxilin and RME‐8 linked to early onset and familial forms of the disease (Edvardson et al. 2012; Vilariño‐Güell et al. 2014). Both chaperones function in the clathrin uncoating of SVs, with Auxilin binding assembled clathrin coats (Park et al. 2015; Yim et al. 2010) and RME‐8 associating with synaptic endosomes and facilitating clathrin coat dynamics at this presynaptic organelle (Xhabija and Vacratsis 2015).

Thus, evidence from the field of molecular chaperone function at the presynaptic terminal further shows the complex interplay between molecules chaperoning SV trafficking and neurodegenerative disease, further highlighting the importance of tight control of SV trafficking and synaptic homeostasis.

### 6.5 Synaptic Autophagy and Neurodevelopmental Disorders

Neurodevelopmental‐associated genetic mutations are often part of the autophagy pathway (for a review see Deng et al. 2021). Whole exome sequencing data from individuals with autism spectrum disorders (ASDs) allowed the identification of copy number variation (CNV) for multiple genes in the autophagy pathways: *GABARAPL2, GABARAPL1, MAP1LC3A, GABARAP*, and *MAP1LC3B* (Poultney et al. 2013). Mutations in several key regulators of autophagy have been associated with global developmental delay and human neurodevelopmental disorders, such as *WIPI2, VPS15* and *ATG7* (Collier et al. 2021; Gstrein et al. 2018; Jelani et al. 2019).

While evidence for a neuronal role of autophagy in ASDs and complex neurodevelopmental disorders is strong, there is less evidence to show a localised effect of autophagy on the synaptic terminal. However, autophagy itself plays critical roles in the development of the nervous system and synaptogenesis (Shen and Ganetzky 2009), with altered synaptogenesis frequently seen in neurodevelopmental disorders.

#### 6.5.1 Lysosomal Storage Disorders

Synaptic dysfunction is also an early and prominent feature of neurological lysosomal storage disorders (LSDs). LSDs are a set of progressive, young‐onset conditions which share overlapping features of neurodevelopmental and neurodegenerative conditions and lead to the characteristic deposition of autofluorescent autophagolysosomes in cells.

The LSD mucopolysaccharidosis type IIIA (MPS‐IIIA) is caused by deficiency in a lysosomal hydrolase (Valstar et al. 2010). Ultrastructural analysis of the presynaptic terminals of MPS‐IIIA model mice revealed the presence of abnormal vacuoles, and the concomitant decrease per terminal in the number of SVs, correlating with impaired synaptic function (Sambri et al. 2017). Similarly, in a mouse model of the LSD neuronal ceroid lipofuscinosis caused by mutation in the lysosomal hydrolase palmitoyl protein thioesterase (PPT1), decreased numbers of SVs per terminal were also seen in cultured cortical neurons (Virmani et al. 2005). This evidence further strengthens the complex balance between endolysosomes, autophagy and SV number, and the knock‐on effect on the availability of SV pools for sustained neurotransmission.

## 7 Discussion

It is abundantly clear that synaptically localised mechanisms of proteostasis are frequently dysregulated in neurodevelopmental and neurodegenerative conditions (Figure 1).

### 7.1 Postsynaptic Roles for Protein Degradation Pathways

Given the multiple points of interplay among the complex presynaptic membrane trafficking pathways of SV turnover, endolysosomal and autophagosome biogenesis, and trafficking, this review focuses primarily on evidence for presynaptic deficits in autophagy. However, there are also clear physiological postsynaptic roles for endolysosomal trafficking and autophagy, which need to be considered when studying synaptic proteostasis across the spectrum of neurodegenerative and neurodevelopmental disorders.

Postsynaptic autophagy is often activated in response to synaptic activity, such as long‐term depression (LTD) (Compans et al. 2021; Daskalaki et al. 2022; Kallergi et al. 2022). Induction of LTD by activation of NMDA receptors or metabotropic glutamate receptors results in the rapid formation of autophagic structures in the dendrites of cultured neurons and hippocampal slices, and inhibition of autophagic vesicle biogenesis prevents NMDAR‐ and mGluR‐LTD (Daskalaki et al. 2022; Kallergi et al. 2022). NMDA‐dependent LTD was also found to be dependent on the autophagy‐mediated loss of the postsynaptic protein PSD‐95 (Compans et al. 2021).

### 7.2 Non‐Cell Autonomous Mechanisms

The focus here for this review is on cell autonomous location‐specific signalling; however, cell non‐autonomous mechanisms are also incredibly important to consider when identifying potential therapeutic avenues. For example, although inhibiting autophagy by conditionally knocking out ATG7 in the mutant SOD1 mouse motor neurons exacerbates early NMJ pathology (Rudnick et al. 2017), it was also paradoxically found to extend the mouse lifespan. This protective effect was due to non‐cell autonomous mechanisms; reducing autophagy led to reduced glial inflammation and blocked activation of the stress‐related transcription factor c‐Jun (Rudnick et al. 2017).

### 7.3 Translation Fidelity

Accuracy of protein synthesis is dependent on stringent translation fidelity. Errors during the elongation phase of protein synthesis can produce truncated or misfolded protein products. Such errors can arise due to frameshifting, incorrect amino acid incorporation, premature termination, or readthrough of stop codons. Protein production accuracy decreases with age, with translation fidelity found to decline significantly upon ageing in an organ‐dependent manner in mice; for example, stop codon readthrough increases by 50% in the ageing mouse brain (Böttger et al. 2025). Similarly, inducing errors in translation fidelity is associated with accelerated ageing. If error‐prone translation is enhanced by reducing translation fidelity, mice exhibit premature ageing‐associated features (Shcherbakov et al. 2022), showing that intrinsic cellular health is linked to the accuracy of protein synthesis.

Evidence for a role of translation fidelity in neurons comes from studies on tRNA synthetases. Mischarged tRNAs (those loaded with the incorrect amino acid) are normally cleared by the editing function of aminoacyl‐tRNA synthetases. If mischarged tRNAs are not cleared, the incorrect amino acid can be incorporated into a nascent polypeptide during protein production, leading to an aberrant amino acid sequence in a protein. An editing defect in a single tRNA synthetase, the alanyl‐tRNA synthetase gene *Aars*, compromises tRNA proofreading, leading to protein misfolding, upregulation of the unfolded protein response, and cerebellar Purkinje cell loss in mice (Lee et al. 2006).

Another crucial contributor to the correct functioning of newly translated proteins is the correct conformational structure. To perform their intended cellular function, newly folded proteins must adopt the correct 3D conformation. Molecular chaperones are a diverse group of proteins that ensure the correct 3D conformational state of other proteins in the cell. In addition to their above‐discussed roles in maintaining correct function in SV trafficking, molecular chaperones also play a key role in translation fidelity in the canonical, cytosolic production of protein, where they facilitate the stabilisation of nascent peptides, the regulation of ribosome movement, and prevent frame‐shifting or premature termination (for a review see (Topf 2026)).

However, despite the clear importance of each of these stages of translation fidelity in maintaining cellular health, strikingly little is known about the synaptic‐specific aspect of this process in humans. Thus, the relative contribution of translation fidelity to the locally translated protein, and how this differs in disease, is yet unknown. As the single greatest risk factor for neurodegeneration is age, and age itself is coupled to reduced translation fidelity, a better understanding of the fidelity of local protein translation is necessary to further our understanding of disease.

### 7.4 Limitations in the Field

While the importance of understanding synaptically localised pathways of protein translation and degradation is clear, numerous current limitations in the field constrain our understanding of the molecular mechanisms that govern localised proteostasis. Single‐cell transcriptomics has been incredibly beneficial for understanding gene expression stochasticity and providing disease insight with a view to personalised medicine. However, the synaptic terminal is intractable to single‐cell sequencing technologies‐ during the sorting for single‐cell sequencing, the long and fragile neuritic projections of the dendrites and axons are sheared, and the protein and RNA populations from these distal projections are thus lost from analysis. Researchers have been innovating novel techniques to circumvent this loss, with interesting approaches such as axon‐seq (Nijssen et al. 2018), synaptosomal purifications (Bernard et al. 2022), and expansion microscopy (Hafner et al. 2019) giving significant insight into the microenvironment of the axon and synaptic compartments.

#### 7.4.1 Selective Neuronal Vulnerability

A question that has not been adequately answered in the field and deserves more focused attention is the selective vulnerability of certain neuronal subtypes. For example, while it's clear that ALS and FTD exist on a disease spectrum, it's not yet known why mutations in certain genes lead to the loss of motor neurons in ALS, while mutations in other genes will lead to loss of neurons in the frontal and temporal lobes in FTD. Even more puzzling is the fact that the same mutation in the same gene (e.g., the hexanucleotide repeat expansion in *C9orf72*) can lead to either ALS or FTD‐ even within the same family (Savica et al. 2012). Clearly there are environmental and genetic risk factors at play that we do not yet know, which predispose neuronal subtypes differently. As synaptic dysfunction is so intrinsically important in disease, further definition of the molecular mechanisms underlying synaptic dysfunction is imperative to further our development of meaningful therapeutics.

#### 7.4.2 Synaptic‐Specific Rescue of Deficits?

Another question that the field needs to explore is how to achieve a subcellularly localised rescue of synaptopathy; a synaptic‐specific rescue of proteostasis imbalance. Modulation of autophagy has long been proposed as a treatment for both neurodegenerative and neurodevelopmental disorders, such as FXS. However, this approach to date has produced limited efficacy and significant side effects. For example, treating *Fmr1* KO mice chronically with an mTORC1 inhibitor, rapamycin, to upregulate autophagy did not rescue the behavioural phenotypes in the KO animals and, in fact, actually had an adverse effect on sleep and social behaviour in both *Fmr1* KO and the control mice (Saré et al. 2017). A more nuanced approach would be to achieve compartment specific modulation of proteostasis‐ achieving this compartment specific modulation to treat the initial site of dysfunction at the synapse offers a radical way to treat synaptopathy without unwanted off‐target side effects. Better understanding of how local protein translation and degradation is controlled at the synaptic terminal will allow novel innovative ways to achieve this.

## 8 Conclusion

The links between proteostasis and progressive neuron dysfunction and degeneration are numerous, highlighting the continued importance of studying these pathways to further our understanding of synaptic physiology and pathophysiology. As the synapse is an early site of dysfunction, it offers a unique opportunity as a site for therapeutic intervention. With the inherited forms of neurodegenerative disease, having a synaptic therapeutic that could mitigate the dysfunction caused by altered proteostasis at an early point before neuron loss could potentially prevent progression of the disease. Indeed, even more excitingly, as more disease biomarkers are being developed, we are at a promising point in medical development where biomarker detection and diagnosis of disease could allow synaptic therapeutics to be targeted to sporadic cases at an early stage in disease onset. Better understanding of the molecular mechanisms occurring in synaptopathies is thus incredibly important in order to realise these exciting possibilities.

## Author Contributions

**Paul G. Donlin‐Asp:** visualization, writing – original draft, writing – review and editing. **Emma L. Clayton:** visualization, writing – original draft, writing – review and editing.

## Funding

P.G.D.‐A. is funded by the Simons Initiative for the Developing Brain (SIDB), a research grant from CureSynGAP1, and a Wellcome Trust Career Development Award (309570/Z/24/Z). E.L.C. is funded by an Alzheimer's Research UK Senior Fellowship [ARUK‐SRF2022A‐005], and the Alzheimer's Society (571).

## Data Availability

The authors have nothing to report.
